# Supplementary material for: Dynamin-dependent entry of Chlamydia trachomatis is sequentially regulated by the effectors TarP and TmeA
Source: Nat Commun. 2024 Jun 10;15:4926. doi: 10.1038/s41467-024-49350-6 (PMC11164928; doi:10.1038/s41467-024-49350-6)
Supplement: Supplementary file 1 — Supplementary Information [file 41467_2024_49350_MOESM1_ESM.pdf]

Supplemental Figure 1

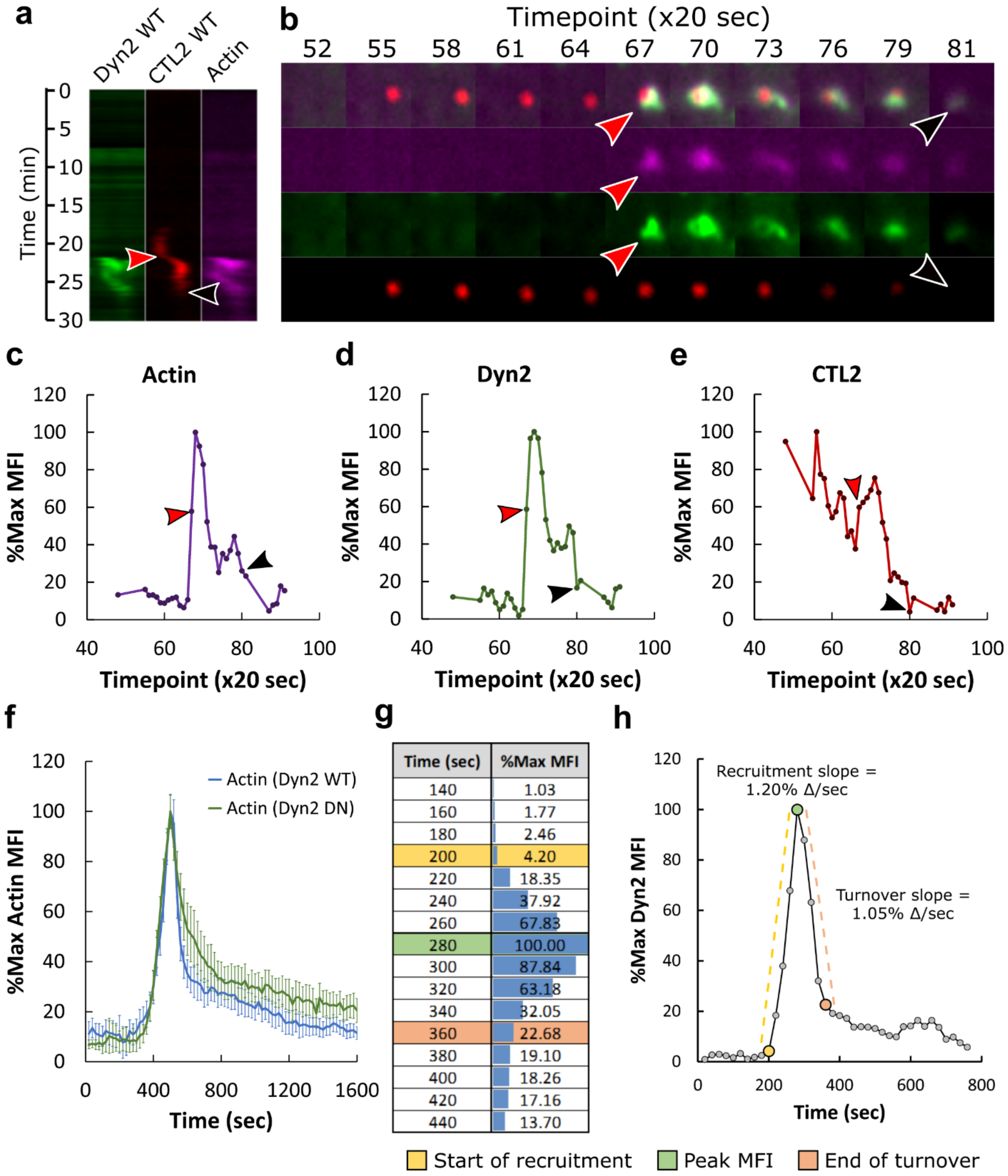

Supplemental Figure 1: Strategy for detailed analysis of protein recruitment at *Chlamydia* entry sites.

(A-E) Cos7 cells were transfected with GFP-Dyn2 WT and iRFP670-LifeAct for 24 hours prior to infection with red fluorescent CMTPX-*Chlamydia* wild-type (MOI = 20). Infection was monitored by live-cell confocal microscopy, acquiring images in 20s intervals. (A) Fluorescent signal obtained from Dyn2 (GFP/green), *Chlamydia* (RFP/red), and actin (Far-red/magenta) channels over a 30-minute time course were depicted as a kymograph. (B) Quantification of *Chlamydia* internalization duration was conducted by identifying the timepoint in which protein recruitment began (red arrow), representing the start of internalization, and the timepoint in which *Chlamydia* signal disappeared (black arrow), representing entry of pathogen into host cells. Internalization duration is defined as time elapsed between initiation of protein recruitment and pathogen entry into host cells. (C,E) Fluorescence intensity from actin (C), Dyn2 (D), and *Chlamydia* (E) was normalized as percent maximal intensity for each timepoint and plotted on a line graph, demonstrating that actin and Dyn2 recruitment is concomitant. (F) Recruitment events derived from live-cell imaging experiment described above (A) were quantified as described in Fig. 1C and plotted as %max actin MFI for each timepoint +/- SEM, comparing actin recruitment between cells expressing GFP-Dyn2 WT or GFP-Dyn2 DN alongside iRFP670-LifeAct. Graphs depict a minimum N=18 recruitment events per treatment/experimental group. (G-H) Kinetic analysis of host protein recruitment within *Chlamydia* entry sites was conducted by calculating the slope representing percent rate of change (%Δ) per second for each recruitment event. (H) Start of recruitment = first timepoint exhibiting MFI above background, Peak MFI = timepoint with 100% (max) MFI, End of turnover = restoration of basal fluorescence intensity. Lingering fluorescent signal is present after the end of turnover, which dissipates slowly (5-10+ min) and non-uniformly across recruitment events and is omitted from analysis. Protein recruitment rates reported in violin plots throughout the study are defined as the slope between the start of recruitment (yellow) and peak MFI (green), while turnover rates are defined as the slope between peak MFI (green) and end of turnover (orange). Quantification of internalization duration, recruitment and turnover rates were calculated for each independent recruitment event and plotted onto violin plots found throughout the study.

Supplemental Figure 2

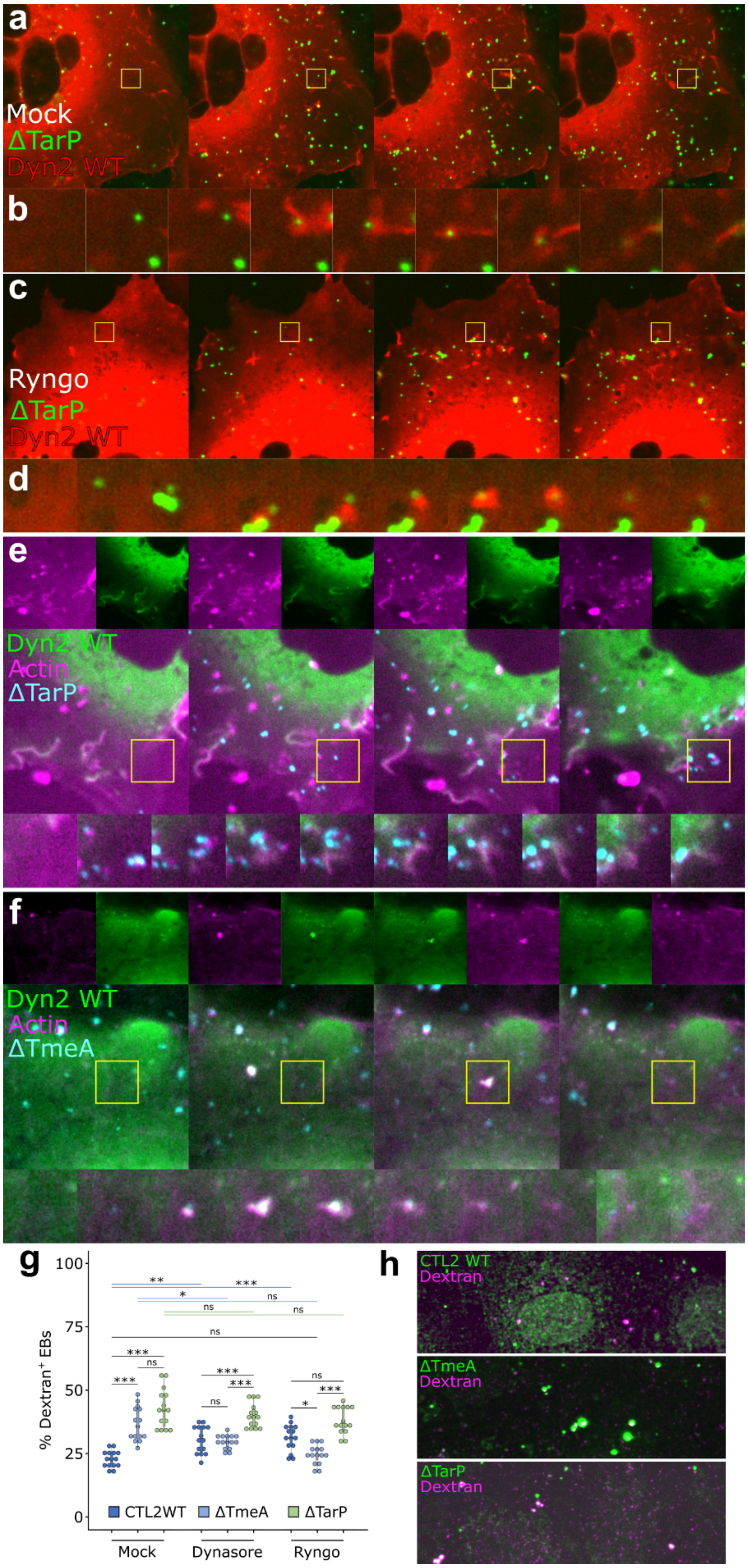

29

Supplemental Figure 2: TarP deletion mutants achieve uptake via ruffles enriched with Dyn2 and actin.

Cos7 cells were transfected with RFP-Dyn2 WT alone (A-D) or alongside iRFP670-LifeAct (E,F) for 24 hours prior to infection with GFP-ΔTarP (A-E) or CMFDA-ΔTmeA (F) EBs (MOI=20) in the absence (A,B,E,F) or presence (C,D) of 40 µM Ryngo 1-23. Uptake of ΔTarP and ΔTmeA EBs was monitored by live-cell confocal microscopy, obtaining images once every 20 seconds for 30 minutes. (A) Montage of mock-treated, ΔTarP infected Cos7 cells expressing RFP-Dyn2 WT, highlighting assembly of Dyn2-rich ruffles in the vicinity of invading bacteria. Yellow box indicates cell region used in (B) to generate representative montage depicting ΔTarP EB uptake. (C) Montage of Cos7 cells expressing RFP-Dyn2 WT, pretreated with 40 µM Ryngo 1-23 for 30 minutes prior to infection with ΔTarP EBs, highlighting the localized recruitment of RFP-Dyn2 at ΔTarP entry sites. Yellow box indicates cell region used in (D) to illustrate uptake of ΔTarP EBs following localized Dyn2 recruitment. (E) Montage of Cos7 cells co-expressing both RFP-Dyn2 WT and iRFP670-LifeAct for 24 hours prior to infection with GFP-ΔTarP EBs (MOI=20), demonstrating that ruffles are enriched with both Dyn2 and actin. (F) Montage of Cos7 cells co-expressing both RFP-Dyn2 WT and iRFP670-LifeAct for 24 hours prior to infection with CMFDA-ΔTmeA EBs (MOI=20), demonstrating that Dyn2 and actin are co-recruited immediately at sites of host/pathogen interaction. (G,H) Cos7 cells were treated with 25 µM Dynasore, 40 µM Ryngo 1-23 or DMSO in media containing 100 µg/mL Dextran Alexa Fluor 647 (10kDa) and incubated at 37°C for 30 minutes. Cells were infected with the indicated strain (MOI=50), synchronizing infection by sedimentation at 4°C for 30 minutes before addition of prewarmed media, allowing infection to proceed for 20 minutes at 37°C prior to fixation and staining with anti-MOMP antibody. (G) Cells were analyzed by confocal microscopy, quantifying the percentage of EBs which colocalize with fluorescent dextran, reporting data as a dotplot with inset boxplot depicting median value and interquartile range. Statistical significance was determined by pairwise T-test with Bonferroni post-correction (H) Representative fields depicting colocalization of fluorescent *Chlamydia* and dextran.

Supplemental Figure 3

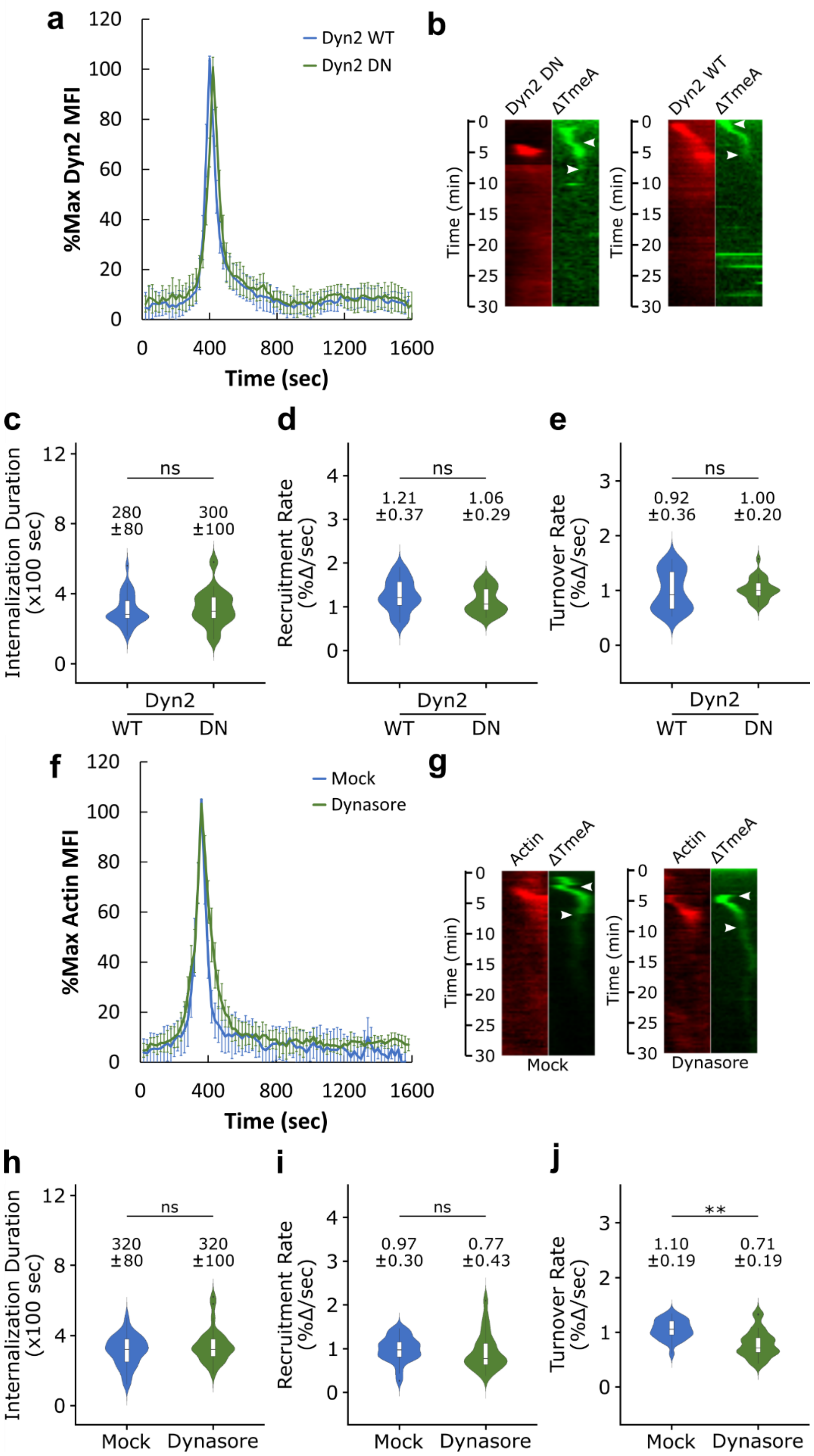

54

Supplemental Figure 3: TmeA-independent uptake is insensitive to Dynamin 2 activity

(A-E) Cos7 cells were transfected with either RFP-Dyn2 WT or RFP-Dyn2 DN for 24 hours prior to infection with green fluorescent CMFDA-stained ΔTmeA EBs (MOI=20). Infection was monitored by live-cell confocal microscopy, collecting images at 20-s intervals. (A) Dyn2 fluorescence intensity at ΔTmeA entry sites was obtained for each timepoint and normalized as %Max MFI independently for each recruitment event. Normalized intensity values were aligned according to the start of Dyn2 recruitment for each event and depicted as line graphs representing the average MFI +/- SEM for each timepoint. (B) Kymographs depicting RFP-Dyn2 and GFP-*Chlamydia* fluorescence over a 30-minute timelapse imaging. Top arrow indicates initiation of protein recruitment and bottom arrow indicates completion of pathogen entry. (C-E) Detailed analysis of each recruitment event obtained via live cell imaging, plotting the (C) internalization duration, (D) rate of Dyn2 recruitment and (E) Dyn2 turnover on violin plots with inset boxplot reporting the median value and interquartile range for each condition. (C) Internalization duration was quantified by calculating the elapsed time between initiation of protein recruitment and termination of pathogen entry, as detailed in Fig. S1. Individual rates of Dyn2 recruitment (D) and turnover (E) were calculated by measuring the slope derived from basal Dyn2 MFI to peak MFI for recruitment, and peak Dyn2 MFI to basal MFI for turnover, as detailed in Fig. S1. Data was obtained from a minimum N=31 individual rates per treatment/experimental group. (F-G) Cos7 cells were transfected with mRuby-LifeAct for 24 hours and pretreated with 25 µM Dynasore for 30 minutes prior to infection with CMFDA-stained ΔTmeA EBs (MOI=20). Infection was monitored and analyzed as described above, reporting the (F) normalized fluorescence intensity of actin recruitment for each timepoint, (G) kymographs of actin and *Chlamydia* fluorescence over a 30 minute timecourse, (H) internalization duration of ΔTmeA entry, and kinetics of (I) recruitment or (J) turnover of actin within ΔTmeA entry sites. Data was obtained from a minimum N=29 individual rates per treatment/experimental group. Statistical significance was determined by two-sided Wilcoxon ranked-sum. All data are representative of 3 independent experiments, * $P \le 0.05$, ** $P \le 0.01$, *** $P \le 0.001$.

Supplemental Figure 4

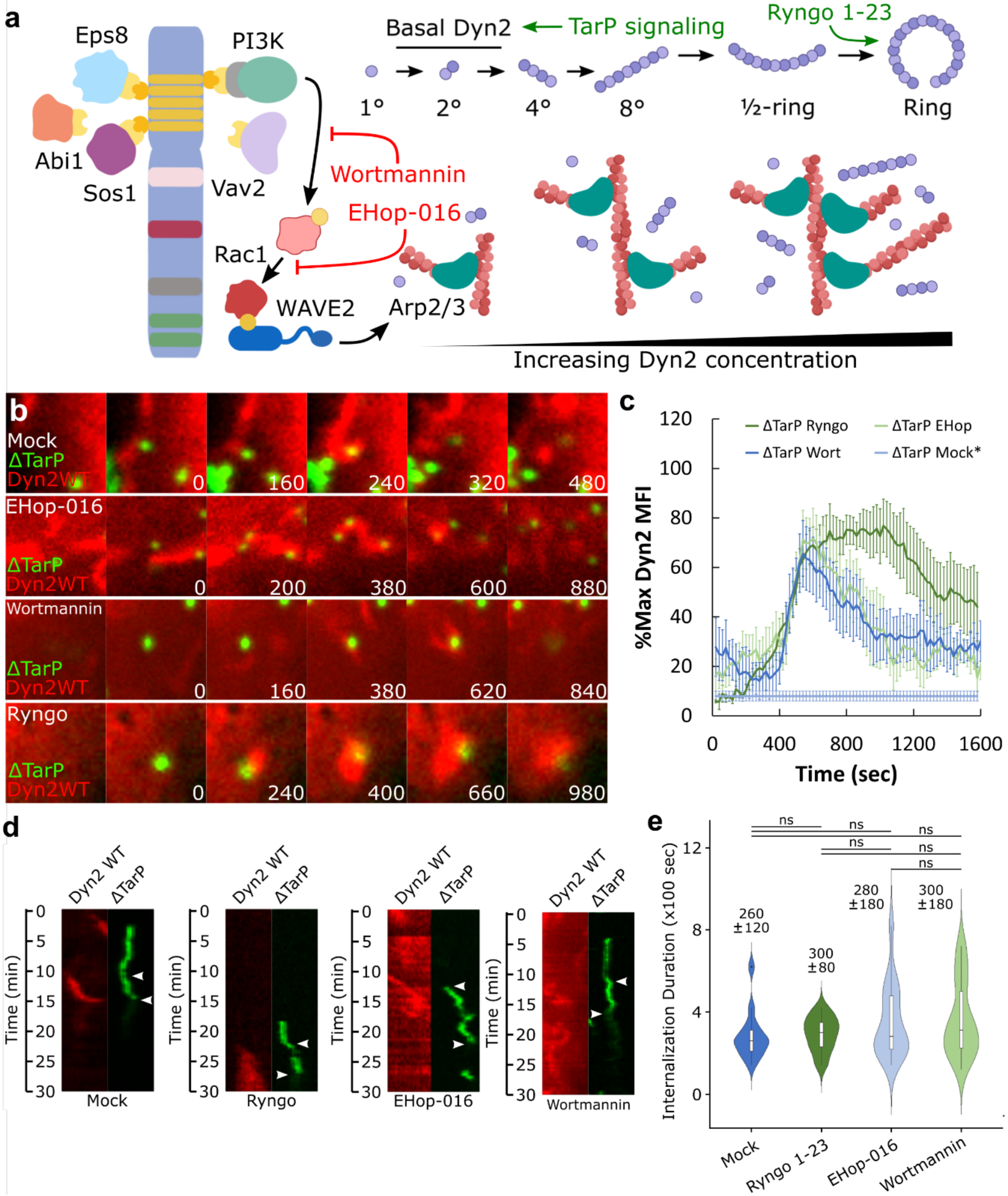

Supplemental Figure 4: TarP-independent uptake does not require Dynamin 2 activity

(A) Schematic depicting TarP signaling via PI3K/Rac1, subsequent recruitment of actin and Dyn2, and Dyn2 oligomerization, highlighting Wortmannin inhibition of PI3K, EHop-016 inhibition of Rac1, promotion of Dyn2 recruitment by TarP, and compound-mediated enhancement of Dyn2 oligomerization by Ryngo 1-

23. (B,C) Cos7 cells were transfected with GFP- or RFP-Dyn2 WT for 24 hours prior to infection with wild-type or ΔTarP EBs at MOI=20 in the presence of 10µM EHop-016, 40nM Wortmannin, 40 µM Ryngo 1-23, or DMSO control. Infection was monitored by live-cell confocal microscopy using a Nikon CSU-W1 spinning disk microscope, obtaining images every 20 seconds for 30 minutes and (B) identifying sites exhibiting Dyn2 recruitment at *Chlamydia* entry sites. Scale bar = 1 micron. (C) Dyn2 recruitment was quantified as described earlier (Fig. 1C) and plotted as %max Dyn2 MFI for each timepoint +/- SEM compiled from a minimum N=17 recruitment events per treatment/experimental group. (D) Kymographs depicting RFP-Dyn2 and GFP-*Chlamydia* fluorescence over a 30 minute timelapse. Top arrow indicates initiation of protein recruitment and bottom arrow indicates completion of pathogen entry. (E) Internalization duration following Dyn2 recruitment was obtained using the same methodology described in Fig. 1E-G. Violin plots contain a minimum N=17 individual events per treatment/experimental group, reporting the median value and interquartile range. Statistical significance was determined by two-sided Wilcoxon Rank-sum. All data are representative of at least 3 independent experiments, * $P \leq 0.05$, ** $P \leq 0.01$, *** $P \leq 0.001$. (*) Placeholder values were utilized for ΔTarP mock recruitment curves (C) as these bacteria invade host cells via a distinct ruffle-mediated entry mechanism, preventing direct comparison with localized Dyn2 recruitment exhibited in other conditions.

Supplemental Figure 5

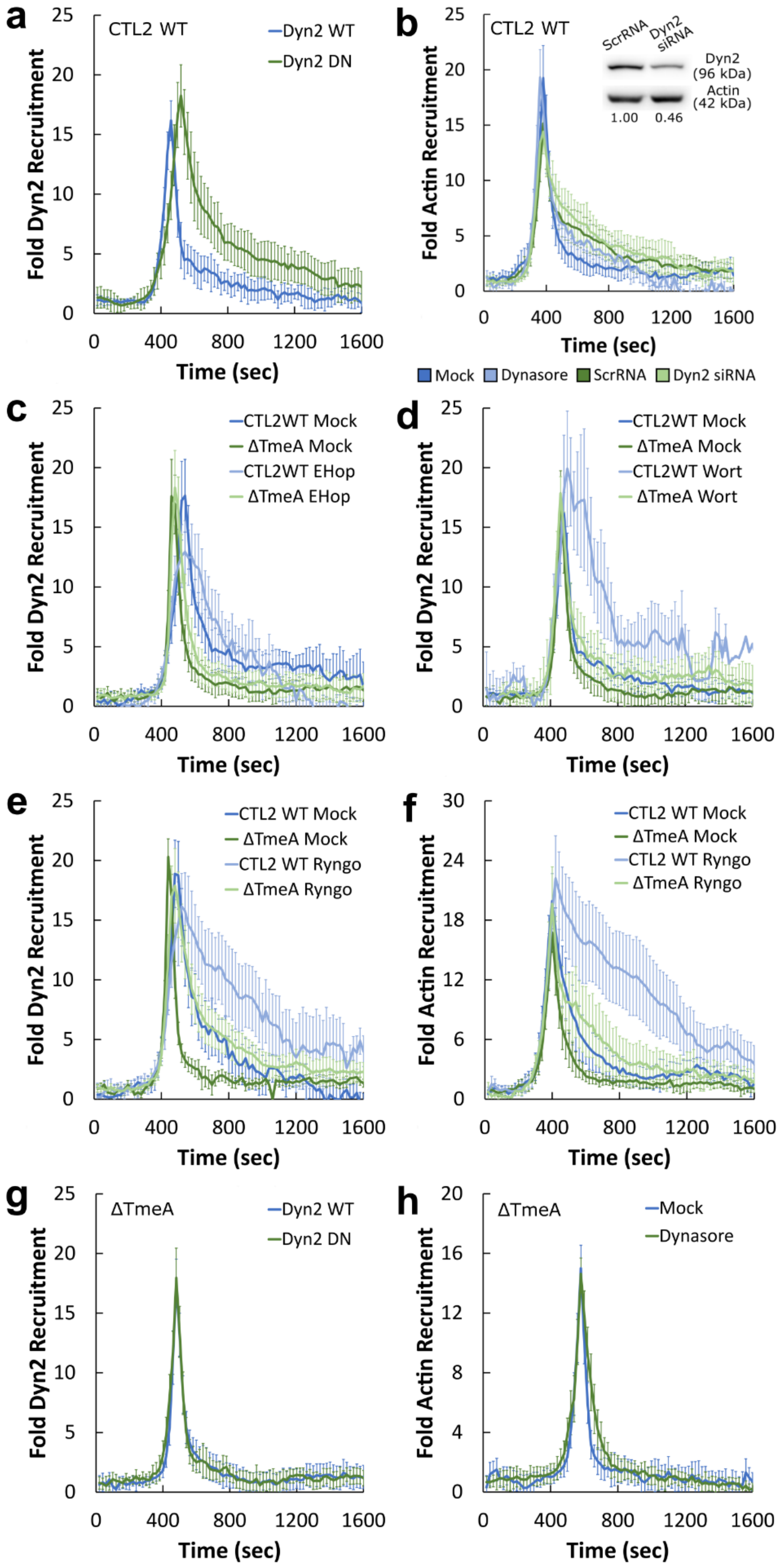

102

Supplemental Figure 5: Recruitment curves of Dyn2 and actin plotting recruitment intensity over time

Cos7 cells were transfected with the indicated protein for 24 hours prior to infection with the indicated *Chlamydia* strain at MOI=20. Infection was monitored by live-cell confocal microscopy using a Nikon CSU-W1 spinning disk microscope, obtaining images every 20 seconds for 30 minutes to identify sites exhibiting actin or Dyn2 recruitment. Background fluorescence was subtracted from protein recruitment sites, and fold recruitment was calculated as a function of the fold increase in mean fluorescence intensity of recruited actin compared to basal actin fluorescence as described previously[7]. Fold recruitment values for graphs depicted in (A) Fig. 1C, (B) Fig. 2B, (C) Fig. 3C, (D) Fig. 4B, (E) Fig. 5D, (F) Fig. 6A, (G) Fig. S3A, or (H) Fig. S3F, each plotted in order to compare the relative intensities of Dyn2 and actin across various conditions.

Figure S6

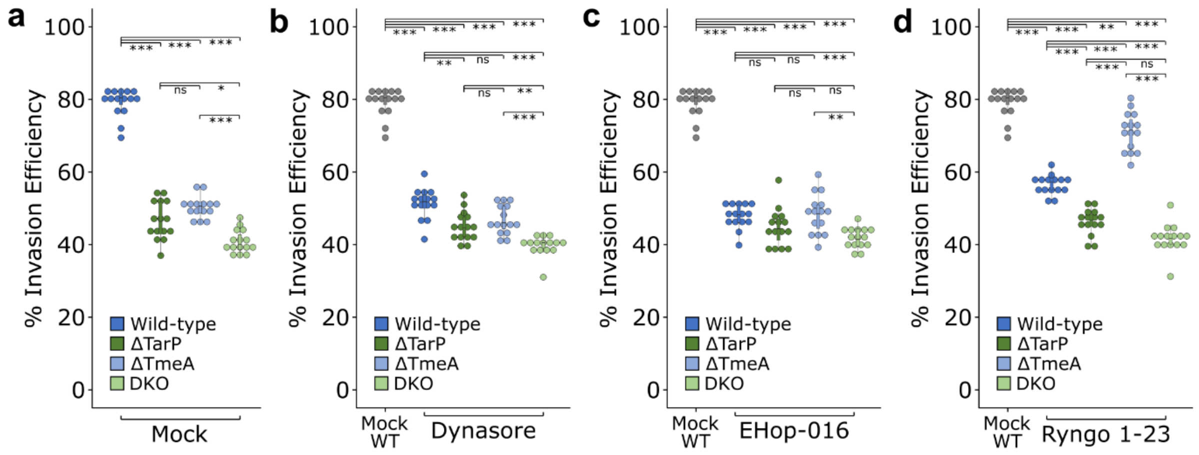

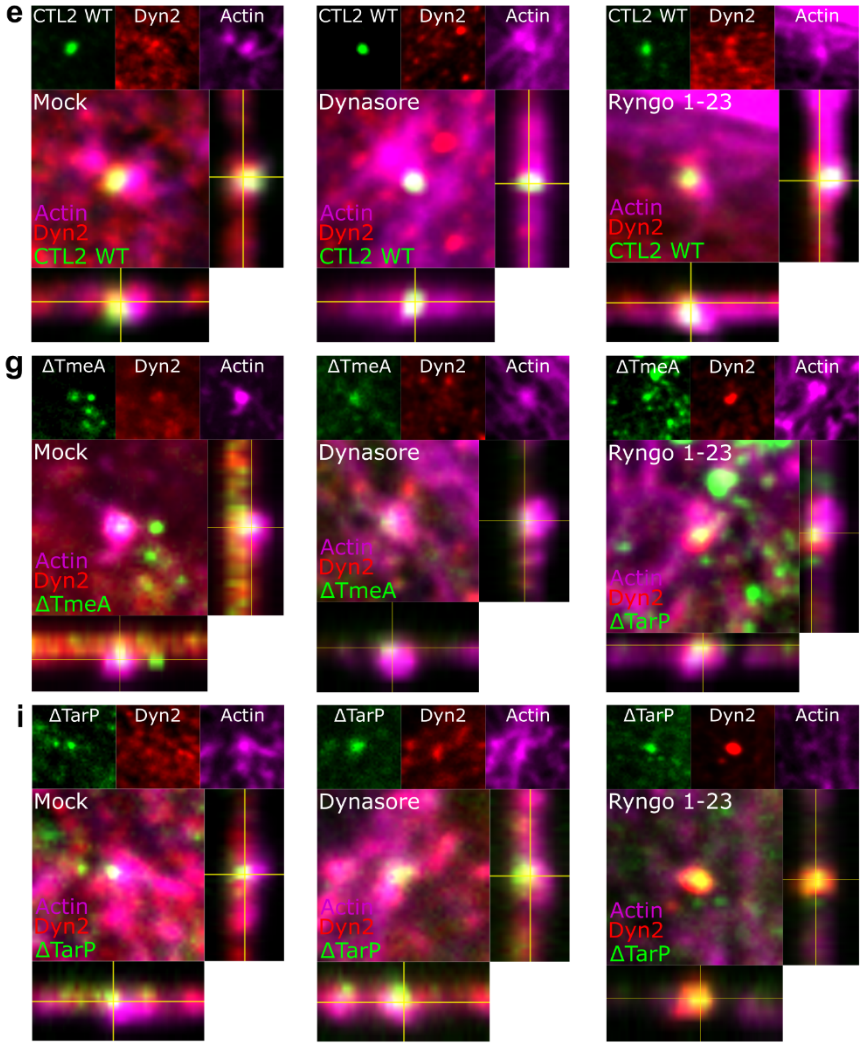

f

CTL2 WT	Mock	Dynasore	Ryngo
%Actin+	85.71	63.16	78.05
%Dyn2+	52.38	47.37	41.46
%Both+	47.62	47.37	41.46

h

ΔTmeA	Mock	Dynasore	Ryngo
%Actin+	60.71	58.82	71.43
%Dyn2+	46.43	47.06	71.43
%Both+	25.00	23.53	52.38

j

ΔTarP	Mock	Dynasore	Ryngo
%Actin+	57.89	61.54	61.29
%Dyn2+	47.37	46.15	51.61
%Both+	36.84	38.46	29.03

Supplemental Figure 6: Dynamin-dependent entry of *Chlamydia* is present in primary human cervical epithelial cells

(A-D) Primary human cervical epithelial cells were infected with the indicated *Chlamydia* strain at MOI=50 and stained using the "in-and-out" method which distinguishes non-internalized EBs from total cell-associated EBs, as described in Materials and Methods. Invasion efficiency was determined for wild-type, ΔTarP, ΔTmeA, or ΔTmeA/ΔTarP (DKO) strains in cells treated with (A) mock DMSO control, (B) 25 µM Dynasore, (C) 10 µM EHop-016, or (D) 40 µM Ryngo 1-23. Data are depicted as dotplots containing inset boxplot reporting median value and interquartile range. Data was collected from 15 fields, with each field containing an average of 50 *Chlamydiae*. (E-J) Primary human cervical epithelial cells were seeded onto coverslips and treated with 25 µM Dynasore, 40 µM Ryngo 1-23, or mock DMSO control for 30 minutes prior to infection with the indicated strain, synchronizing infection by sedimentation prior to incubating infected cell monolayers for 10 minutes. Infected coverslips were stained with mouse anti-MOMP antibody and rabbit anti-Dyn2 antibody, followed by goat anti-mouse Alexa Fluor 488, goat anti-rabbit Alexa Fluor 594, and phalloidin Alexa Fluor 647. Stained coverslips were mounted and imaged, collecting Z-stacks of infected monolayers (0.2 micron step size). Representative events depicting recruitment of actin and Dyn2 at sites of *Chlamydia* entry are shown alongside single-channel images and orthogonal views of the X and Y axes across all Z slices for (E) wild-type, (G) ΔTmeA, or (I) ΔTarP EBs. Proportions of elementary bodies which recruit actin, Dyn2, or both were quantified in an unbiased manner using an automated particle tracking plugin in ImageJ and plotted according to the inhibitor used for (F) wild-type, (H) ΔTmeA, or (J) ΔTarP EBs. Statistical significance was determined by pairwise T- test with Bonferroni post-correction. All data are representative of at least 3 independent experiments, * $P \leq 0.05$, ** $P \leq 0.01$, *** $P \leq 0.001$.

Supplemental Figure 7

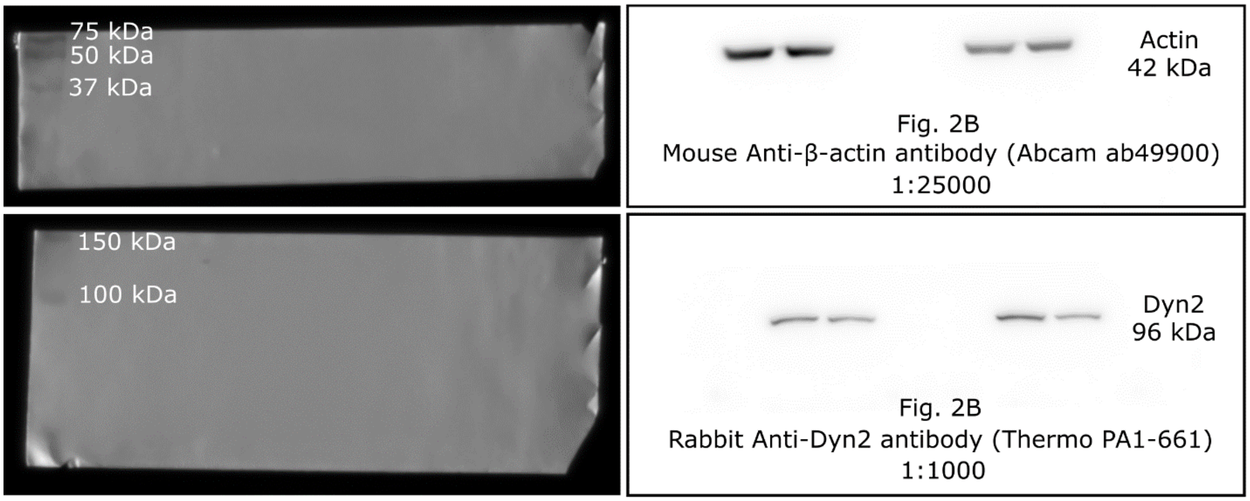

Supplemental Figure 7: Uncropped Western blot used for densitometry measurement to quantify Dyn2 knockdown

Supplemental Video 1: Dynamin 2 recruitment at ΔTarP entry sites in the presence and absence of Ryngo 1-23

Cos7 cells were transfected with RFP-Dyn2 WT for 24 hours prior to infection with ΔTarP EBs at MOI=20 in the presence or absence of 40 µM Ryngo 1-23. Infection was monitored by live-cell confocal microscopy using a Nikon CSU-W1 spinning disk microscope, obtaining images every 20 seconds for 30 minutes.

Supplemental Video 2: Actin recruitment at ΔTmeA entry sites in the presence and absence of Ryngo 1-23

Cos7 cells were transfected with mRuby-LifeAct for 24 hours prior to infection with ΔTmeA EBs at MOI=20 in the presence or absence of 40 µM Ryngo 1-23. Infection was monitored by live-cell confocal microscopy using a Nikon CSU-W1 spinning disk microscope, obtaining images every 20 seconds for 30 minutes. Representative fields are synchronized according to the start of actin recruitment to facilitate comparison of ΔTmeA EB uptake.

Supplemental Video 3: Actin recruitment at wild-type entry sites in the presence and absence of Ryngo 1-23

Cos7 cells were transfected with GFP-Actin for 24 hours prior to infection with wild-type CTL2 EBs at MOI=20 in the presence or absence of 40 µM Ryngo 1-23. Infection was monitored by live-cell confocal microscopy using a Nikon CSU-W1 spinning disk microscope, obtaining images every 20 seconds for 30 minutes. Representative fields are synchronized according to the start of actin recruitment to facilitate comparison of wild-type EB uptake.

Supplemental Video 4: Dyn2 recruitment at wild-type entry sites in the presence and absence of EHop-016

Cos7 cells were transfected with GFP-Dyn2 for 24 hours prior to infection with wild-type CTL2 EBs at MOI=20 in the presence or absence of 10 µM EHop-016. Infection was monitored by live-cell confocal microscopy using a Nikon CSU-W1 spinning disk microscope, obtaining images every 20 seconds for 30 minutes. Videos illustrate reduced Dyn2 recruitment upon inhibition of Rac1 following EHop-016 treatment compared to mock control.

Supplemental Video 5: Dyn2 recruitment at wild-type entry sites in the presence and absence of Wortmannin

Cos7 cells were transfected with GFP-Dyn2 for 24 hours prior to infection with wild-type CTL2 EBs at MOI=20 in the presence or absence of 40 nM Wortmannin. Infection was monitored by live-cell confocal microscopy using a Nikon CSU-W1 spinning disk microscope, obtaining images every 20 seconds for 30 minutes. Videos depict variable Dyn2 recruitment upon inhibition of PI3K following Wortmannin treatment compared to mock control.

Supplemental Video 6: Residual recruitment of actin by invasion-incompetent elementary bodies

Cos7 cells were transfected with GFP-actin or mRuby-LifeAct for 24 hours prior to infection with wild-type CTL2 or ΔTmeA EBs at MOI=20. Infection was monitored by live-cell confocal microscopy using a Nikon CSU-W1 spinning disk microscope, initiating image acquisition 30 minutes after administration of *Chlamydia* EBs to determine the extent of *Chlamydia* internalization at later timepoints. Videos depict that the majority of elementary bodies are inert and incapable of actin recruitment, while others elicit defective and sporadic recruitment of actin.

Supplemental Video 7: Comparison of host protein recruitment dynamics between wild-type, ΔTmeA, and ΔTarP elementary bodies

Cos7 cells were transfected with GFP-actin or mRuby-LifeAct for 24 hours prior to infection with wild-type CTL2, ΔTmeA, or ΔTarP EBs at MOI=20. Infection was monitored by live-cell confocal microscopy using a Nikon CSU-W1 spinning disk microscope, obtaining images every 20 seconds for 30 minutes. Videos depict intense punctate recruitment of actin at the immediate site of *Chlamydia*/host contact for wild-type and ΔTmeA EBs, compared to the diffuse and morphologically distinct actin-rich ruffles which engulf ΔTarP EBs.
