# Supplementary material for: Dynamin-dependent entry of Chlamydia trachomatis is sequentially regulated by the effectors TarP and TmeA
Source: Nat Commun. 2024 Jun 10;15:4926. doi: 10.1038/s41467-024-49350-6 (PMC11164928; doi:10.1038/s41467-024-49350-6)
Supplement: Supplementary file 3 — Description of Additional Supplementary Files [file 41467_2024_49350_MOESM3_ESM.pdf]

Description of Additional Supplementary Files

Supplemental Video 1: Dynamin 2 recruitment at ΔTarP entry sites in the presence and absence of Ryngo 1-23

Cos7 cells were transfected with RFP-Dyn2 WT for 24 hours prior to infection with ΔTarP EBs at MOI=20 in the presence or absence of 40 μM Ryngo 1-23. Infection was monitored by live-cell confocal microscopy using a Nikon CSU-W1 spinning disk microscope, obtaining images every 20 seconds for 30 minutes.

Supplemental Video 2: Actin recruitment at ΔTmeA entry sites in the presence and absence of Ryngo 1-23

Cos7 cells were transfected with mRuby-LifeAct for 24 hours prior to infection with ΔTmeA EBs at MOI=20 in the presence or absence of 40 μM Ryngo 1-23. Infection was monitored by live-cell confocal microscopy using a Nikon CSU-W1 spinning disk microscope, obtaining images every 20 seconds for 30 minutes. Representative fields are synchronized according to the start of actin recruitment to facilitate comparison of ΔTmeA EB uptake.

Supplemental Video 3: Actin recruitment at wild-type entry sites in the presence and absence of Ryngo 1-23

Cos7 cells were transfected with GFP-Actin for 24 hours prior to infection with wild-type CTL2 EBs at MOI=20 in the presence or absence of 40 μM Ryngo 1-23. Infection was monitored by live-cell confocal microscopy using a Nikon CSU-W1 spinning disk microscope, obtaining images every 20 seconds for 30 minutes. Representative fields are synchronized according to the start of actin recruitment to facilitate comparison of wild-type EB uptake.

Supplemental Video 4: Dyn2 recruitment at wild-type entry sites in the presence and absence of EHop-016

Cos7 cells were transfected with GFP-Dyn2 for 24 hours prior to infection with wild-type CTL2 EBs at MOI=20 in the presence or absence of 10 μM EHop-016. Infection was monitored by live-cell confocal microscopy using a Nikon CSU-W1 spinning disk microscope, obtaining images every 20 seconds for 30 minutes. Videos illustrate reduced Dyn2 recruitment upon inhibition of Rac1 following EHop-016 treatment compared to mock control.

Supplemental Video 5: Dyn2 recruitment at wild-type entry sites in the presence and absence of Wortmannin

Cos7 cells were transfected with GFP-Dyn2 for 24 hours prior to infection with wild-type CTL2 EBs at MOI=20 in the presence or absence of 40 nM Wortmannin. Infection was monitored by live-cell confocal microscopy using a Nikon CSU-W1 spinning disk microscope, obtaining images every 20 seconds for 30 minutes. Videos depict variable Dyn2 recruitment upon inhibition of PI3K following Wortmannin treatment compared to mock control.

Supplemental Video 6: Residual recruitment of actin by invasion-incompetent elementary bodies

Cos7 cells were transfected with GFP-actin or mRuby-LifeAct for 24 hours prior to infection with wild-type CTL2 or ΔTmeA EBs at MOI=20. Infection was monitored by live-cell confocal microscopy using a Nikon CSU-W1 spinning disk microscope, initiating image acquisition 30 minutes after administration of *Chlamydia* EBs to determine the extent of *Chlamydia* internalization at later timepoints. Videos depict that the majority of elementary bodies are inert and incapable of actin recruitment, while others elicit defective and sporadic recruitment of actin.

Supplemental Video 7: Comparison of host protein recruitment dynamics between wild-type, ΔTmeA, and ΔTarP elementary bodies

Cos7 cells were transfected with GFP-actin or mRuby-LifeAct for 24 hours prior to infection with wild-type CTL2, ΔTmeA, or ΔTarP EBs at MOI=20. Infection was monitored by live-cell confocal microscopy using a Nikon CSU-W1 spinning disk microscope, obtaining images every 20 seconds for 30 minutes. Videos depict intense punctate recruitment of actin at the immediate site of *Chlamydia*/host contact for wild-type and ΔTmeA EBs, compared to the diffuse and morphologically distinct actin-rich ruffles which engulf ΔTarP EBs.
