# Supplementary material for: Dynamin-dependent entry of Chlamydia trachomatis is sequentially regulated by the effectors TarP and TmeA
Source: Nat Commun. 2024 Jun 10;15:4926. doi: 10.1038/s41467-024-49350-6 (PMC11164928; doi:10.1038/s41467-024-49350-6)
Supplement: Supplementary file 11 — Reporting Summary [file 41467_2024_49350_MOESM11_ESM.pdf]

nature portfolio

Corresponding author(s): Rey A Carabeo

Last updated by author(s): May 1, 2024

# Reporting Summary

Nature Portfolio wishes to improve the reproducibility of the work that we publish. This form provides structure for consistency and transparency in reporting. For further information on Nature Portfolio policies, see our Editorial Policies and the Editorial Policy Checklist.

## Statistics

For all statistical analyses, confirm that the following items are present in the figure legend, table legend, main text, or Methods section.

| n/a | Confirmed | |
|---|---|---|
| ☐ | ☒ | The exact sample size (*n*) for each experimental group/condition, given as a discrete number and unit of measurement |
| ☐ | ☒ | A statement on whether measurements were taken from distinct samples or whether the same sample was measured repeatedly |
| ☐ | ☒ | The statistical test(s) used AND whether they are one- or two-sided<br>*Only common tests should be described solely by name; describe more complex techniques in the Methods section.* |
| ☐ | ☒ | A description of all covariates tested |
| ☐ | ☒ | A description of any assumptions or corrections, such as tests of normality and adjustment for multiple comparisons |
| ☐ | ☒ | A full description of the statistical parameters including central tendency (e.g. means) or other basic estimates (e.g. regression coefficient) AND variation (e.g. standard deviation) or associated estimates of uncertainty (e.g. confidence intervals) |
| ☐ | ☒ | For null hypothesis testing, the test statistic (e.g. *F*, *t*, *r*) with confidence intervals, effect sizes, degrees of freedom and *P* value noted<br>*Give P values as exact values whenever suitable.* |
| ☒ | ☐ | For Bayesian analysis, information on the choice of priors and Markov chain Monte Carlo settings |
| ☒ | ☐ | For hierarchical and complex designs, identification of the appropriate level for tests and full reporting of outcomes |
| ☒ | ☐ | Estimates of effect sizes (e.g. Cohen's *d*, Pearson's *r*), indicating how they were calculated |

*Our web collection on statistics for biologists contains articles on many of the points above.*

## Software and code

Policy information about availability of computer code

| | |
|---|---|
| Data collection | NA |
| Data analysis | NA |

For manuscripts utilizing custom algorithms or software that are central to the research but not yet described in published literature, software must be made available to editors and reviewers. We strongly encourage code deposition in a community repository (e.g. GitHub). See the Nature Portfolio guidelines for submitting code & software for further information.

## Data

Policy information about availability of data

All manuscripts must include a data availability statement. This statement should provide the following information, where applicable:

- Accession codes, unique identifiers, or web links for publicly available datasets
- A description of any restrictions on data availability
- For clinical datasets or third party data, please ensure that the statement adheres to our policy

Data supporting the findings of this manuscript are available within the article. Raw Data files including raw imaging data, source images for analysis, and spreadsheets containing analyzed data have been deposited to Dryad for archival (DOI: 10.5061/dryad.brv15dvh5).

## Research involving human participants, their data, or biological material

Policy information about studies with human participants or human data. See also policy information about sex, gender (identity/presentation), and sexual orientation and race, ethnicity and racism.

| | |
|---|---|
| Reporting on sex and gender | NA |
| Reporting on race, ethnicity, or other socially relevant groupings | NA |
| Population characteristics | NA |
| Recruitment | NA |
| Ethics oversight | NA |

Note that full information on the approval of the study protocol must also be provided in the manuscript.

# Field-specific reporting

Please select the one below that is the best fit for your research. If you are not sure, read the appropriate sections before making your selection.

☒ Life sciences ☐ Behavioural & social sciences ☐ Ecological, evolutionary & environmental sciences

For a reference copy of the document with all sections, see nature.com/documents/nr-reporting-summary-flat.pdf

# Life sciences study design

All studies must disclose on these points even when the disclosure is negative.

| | |
|---|---|
| Sample size | Single replicate for live-cell recruitment assay comprises: 2 wells per condition, 5-10 recruitment events/well, sampling multiple cells per well. At least 20 independent invasion events per treatment/experimental group were monitored. Data reported in study comprised of 3 replicates. Single replicate for invasion assay comprises: 5 fields imaged for each condition, 100 bacteria quantified in each field. Data reported in study comprised of 3 replicates. |
| Data exclusions | No data were excluded from this study |
| Replication | Experiments were repeated at least three times to ensure that data in this study are reproducible. Results from multiple experiments were used to calculate mean values and conduct statistical analyses. All attempts at replication were successful |
| Randomization | Cells were randomly allocated to experimental groups, except in circumstances where fluorescent protein expression is incompatible with certain experimental groups (e.g., GFP protein expression and GFP-expressing or Alexa-488-labeled bacteria). |
| Blinding | Imaging data were quantified in ImageJ or rStudio, unbiased automated image analysis techniques were employed where applicable. Manual quantification was avoided. |

# Reporting for specific materials, systems and methods

We require information from authors about some types of materials, experimental systems and methods used in many studies. Here, indicate whether each material, system or method listed is relevant to your study. If you are not sure if a list item applies to your research, read the appropriate section before selecting a response.

## Materials & experimental systems

| n/a | Involved in the study | |
|---|---|---|
| ☐ | ☒ | Antibodies |
| ☐ | ☒ | Eukaryotic cell lines |
| ☒ | ☐ | Palaeontology and archaeology |
| ☒ | ☐ | Animals and other organisms |
| ☒ | ☐ | Clinical data |
| ☒ | ☐ | Dual use research of concern |
| ☒ | ☐ | Plants |

## Methods

| n/a | Involved in the study | |
|---|---|---|
| ☒ | ☐ | ChIP-seq |
| ☒ | ☐ | Flow cytometry |
| ☒ | ☐ | MRI-based neuroimaging |

## Antibodies

| | |
|---|---|
| Antibodies used | Antibodies used in this study are as follows: mouse monoclonal anti-MOMP (Novus Biologicals, #NB10066403), rabbit polyclonal anti-Chlamydia trachomatis (Abcam ab252762), Alexa Fluor 594 anti-mouse (ThermoFisher #A11032), Alexa Fluor 488 anti-rabbit (ThermoFisher #A11034), rabbit polyclonal anti-Dyn2 (Thermo PA1-661), Alexa Fluor 488 anti-mouse (ThermoFisher # A-11001), Alexa Fluor 594 anti-rabbit (ThermoFisher # A-11012), HRP Anti-beta Actin antibody [AC-15] (Abcam ab49900) |
| Validation | All antibodies are commercially sourced and used according to manufacturer's directions without further validation. |

## Eukaryotic cell lines

Policy information about cell lines and Sex and Gender in Research

| | |
|---|---|
| Cell line source(s) | Green monkey kidney fibroblast-like (Cos7) cells, cervical adenocarcinoma epithelial (HeLa) cells, and McCoy B mouse fibroblasts originate from (Carabeo et al., 2004). McCoy B mouse fibroblasts were originally obtained from Dr. Harlan Caldwell, NIH/NIAID. Primary human cervical epithelial cells (HCECs, ATCC PCS-0480-011, Lot 80306190) were purchased from ATCC. |
| Authentication | None of the cell lines used were authenticated |
| Mycoplasma contamination | Mycoplasma testing was routinely conducted using the ATCC universal mycoplasma detection kit (ATCC 30-1012K). All cell lines are confirmed mycoplasma negative |
| Commonly misidentified lines (See ICLAC register) | HeLa cells were utilized in this study to quantify Chlamydia invasion efficiency. Chlamydia trachomatis promiscuously infects cultured cells and yields consistent invasion efficiency results across cultured cell lineages. HeLa cells were utilized due to ease of culture and its cervical lineage. |

## Plants

| | |
|---|---|
| Seed stocks | NA |
| Novel plant genotypes | NA |
| Authentication | NA |
