# Supplementary figures and images for: Dynamin-dependent entry of Chlamydia trachomatis is sequentially regulated by the effectors TarP and TmeA
Source: Nat Commun. 2024 Jun 10;15:4926. doi: 10.1038/s41467-024-49350-6 (PMC11164928; doi:10.1038/s41467-024-49350-6)

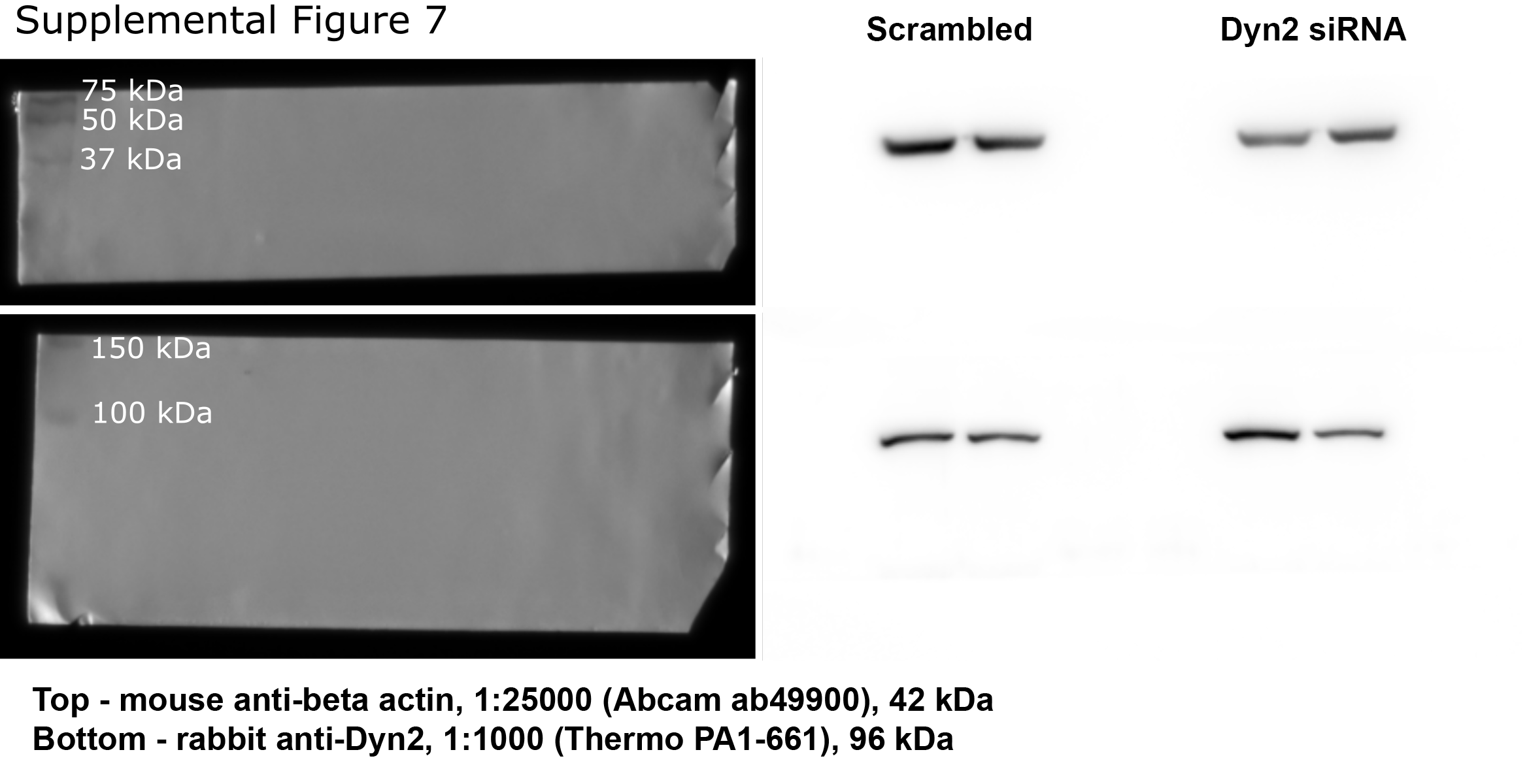

Supplement: Supplementary file 12 — Source data [file 41467_2024_49350_MOESM12_ESM.zip › FigS7 v2.png]
